# Clinical Study of Graft Selection in Malaysian Rhinoplasty Patients

**DOI:** 10.1155/2013/639643

**Published:** 2013-09-03

**Authors:** Balwant Singh Gendeh

**Affiliations:** Department of Otorhinolaryngology-Head Neck Surgery, UKM Medical Center, Jalan Yaacob Latif, Bandar Tun Razak, 56000 Kuala Lumpur, Malaysia

## Abstract

Graft selection remains the greatest challenge for surgeons performing rhinoplasty. The preferred choice thus far for nasal reconstruction would be autograft compared to allograft due to its lower rate of infection and extrusion as it does not induce an immune response. We have evaluated 26 patients who underwent open structured rhinoplasty at our center and compared our experience regarding the operative technique, graft availability, indications, and limitations. The racial distribution was 18 Indians, 5 Chinese, and 3 Malays with a mean age, hospitalization, and followup of 30.5 years, 16.9 months, and 4.4 days, respectively. Majority of the patients (57.6%) presented with twisted nose and 30.7% of the patients presented with history of nasal trauma. All the patients had deviated septum of varying severity. The most common graft used was quadrangular cartilage graft and the common complications noted were ala deformity and tip anaesthesia in 7.6% patients respectively.

## 1. Introduction

In multiethnic Malaysian population, the nasal profile and skin thickness in the Oriental Chinese and Malay noses vary from the Caucasian Middle Eastern and Indian noses. Augmentation rhinoplasty is more common in Oriental Chinese or Malay noses whereas reduction rhinoplasty is more common in Middle Eastern and Indian noses.

Rhinoplasty is an operation planned to reshape the anatomic features of the nose into a new more pleasing relationship with the surrounding facial features. Rhinoplasty consists of septoplasty, tip remodeling, hump removal, narrowing of nose with osteotomies, and final correction of subtle deformities. The results achieved in rhinoplasty are directly related to the surgeon's ability to elucidate how subtle change in the bony and cartilaginous support of the nose will change its appearance [1]. 

Numerous grafting techniques have been developed to sculpt the nasal framework in rhinoplasty over time. These basic techniques have evolved from the principal that maintenance of major supporting structures of the nose is fundamental for aesthetic and functional purposes. However, the type of graft, its shape, position, and usage may vary depending on the situation and the objectives of the surgeon. Despite the advances and the multiple techniques that have been described in the literature, it can be a steep learning curve and a daunting task for the aspiring rhinoplasty surgeon. The surgeon's attention to functional, reconstructive, and aesthetic principles is paramount in ensuring optimum septorhinoplasty results, much to the satisfaction of both the patient and the surgeon.

In a twisted nose, whether posttraumatic or nontraumatic, the nasion and nasal tip are in the same vertical plane with the midvault deviated to one side. On the contrary, in a crooked nose, the nasion, mid-vault, and the nasal tip are in a straight line off the vertical plane. A saddled nose, owing to the loss of the dorsal aspect of the quadrangular cartilage, has a supra tip depression, shortening of nose, and often overrotation of the tip. The nasal tip can also be deformed in many ways—overprojected, underprojected, rotated, and abnormally shaped, amongst others. Nasal deformities and alar asymmetry are most significant in cleft lip/cleft palate patients as compared to traumatic or nontraumatic nasal deformity patients.

New techniques are formulated with greater attention to the nasal anatomic variability in humans across different ethnic groups. Proponents of the closed or endonasal approach emphasize on its advantages, namely, absence of external incisions and less dissection required, therefore minimizing soft tissue trauma and subsequent scarring. It is less dependent on postoperative steroids to reduce postoperative swelling. However, exposure to the surgical field is very limited, and tip supporting mechanism tends to be compromised with time [2].

On the contrary, the open or external approach offers a much superior exposure of the nasal tip for inspection of the nasal osteocartilaginous framework without anatomic distortion, therefore allowing proper remodeling of the nasal framework. The surgeon can be assured of accuracy while performing detail suturing and resection manipulation. It also offers unparallel accuracy for structural diagnosis and placement or manipulation of graft, if needed, under direct vision. Being a tertiary referral hospital, the author's preference for open approach also facilitates the teaching and learning of nasal anatomy and surgical techniques especially for revision or secondary septorhinoplasty. On the other hand, opponents argued that the transcolumellar incision used for surgical access in this technique produces scarring [3]. 

The author, being a proponent of open septoplasty approach, reviewed his surgical patients who have undergone this technique for various reasons.

## 2. Materials and Methods

This is a retrospective review of 26 patients with either developmental or posttraumatic nasal deformities who were treated surgically over a period of 65 months from July 2002 till December 2007 at the UKM Medical Center, Kuala Lumpur. The nasal profile and the skin thickness of the individual racial groups were assessed. Following which, these patients either underwent reduction rhinoplasty, augmentation rhinoplasty, tip plasty, external osteotomies, or rasping of hump with or without corrective septal surgery and inferior turbinate reduction, after a comprehensive consent was obtained. The patient was counseled by the surgeon regarding the postoperative outcome whereby the aim was to achieve both an improvement in nasal function and cosmesis. Patients records were then reviewed concerning graft selection, complications encountered, functional as well as cosmetic improvement based on a symptom score (1–10) and whom required revision procedures.

## 3. Results

The demographic data of all the patients (mean age 30.5 years) who underwent open septorhinoplasty are shown in Table 1. On presentation, the patients were assessed on the complaints of nasal blockage and/or cosmetic inadequacies. A detailed history was obtained, and physical examination and nasal endoscopy were performed. Photographs for preoperative documentation were taken from 5 views: frontal, basal, top, right, and left 45 degree oblique. Nasal deformity on presentation can be summarized as in Table 2.

Majority (57.6%) of the patients presented with twisted nose. There were two patients who underwent endonasal septoplasty earlier elsewhere, but the residual deformity in the form of a crooked nose and prominent dorsal hump compelled correction via the open septorhinoplasty approach. There was history of obvious nasal trauma elicited in 8 of the 26 (30.7%) patients. All the patients had deviated nasal septum of varying severity.

### 3.1. Operative Technique

All the patients were orotracheally intubated with the tube centrally placed, and the head was slightly extended. With the oropharyngeal pack in place, both nostrils were packed with cotton pledgets soaked in cocaine adrenaline (1 : 1000 concentration) for vasoconstriction. Local anaesthesia in the form of ropivacaine 2 mg/mL and adrenaline 1 : 80,000 was injected into the nasal tip, columella, and nasal septum along the site of proposed marginal incision and along the lateral nasal wall. All cases were approached via a combined, either inverted *V*- or *Z*-shaped, transcolumellar incision with bilateral alar marginal incisions using a size-15 scalpel blade. The marginal incision along the caudal margin of the lateral crura was extended down to the columella to meet the columellar incision. With the aid of an Aufricht Retractor and small curved scissors, the soft tissue plane was dissected below the superficial muscular aponeurotic system (SMAS) superiorly and laterally to expose the upper lateral cartilage and the lateral crura, respectively. The middle nasal vault was exposed in the midline. The interconnecting ligaments over the medial crura were split exposing the caudal portion of the septal cartilage. Bilateral mucoperichondrial flap of the cartilaginous septum was elevated with the dissection continuing over the perpendicular plate of the ethmoid bone and vomer upward and extending over the nasal crest of the maxillary bone and medial floor of the nose downward. After completion of the degloving exposure, bipolar electrocautery was used for hemostasis. The entire osteocartilaginous framework was then elevated.

Suitable grafts in the form of autograft (septal bone, conchal cartilage, quadrangular cartilage spreader grafts, and rib cartilage graft) or allograft (porous high-density polyethylene—Medpor, Porex Surgical, Georgia, USA) were placed in the area of the defect. The types of grafts used for various nasal deformities are shown in Table 1. Autogenous grafts in the form of spreader graft from the septal cartilage were the graft of choice used in 14 (53.8%) patients. In 7 (26.9%) patients, more than one graft material was used, namely, as quadrangular cartilage spreader graft and medpor (dorsal support graft or spreader graft) and rib cartilage graft (spreader graft, dorsal graft, columella graft, shield graft, and baton graft).

The patients with underprojected tip and saddled nose had tip recontouring performed to elevate the nasal tip. On completion, the skin flap was returned to its normal anatomical position, and the transcolumellar incision closed with nylon 5/0 sutures while the bilateral alar marginal incision with Vicryl 4/0 sutures.

### 3.2. Postoperative Evaluation

Postoperatively, the patients were prescribed a course of amoxicillin/clvaulanate for five days. Adhesive dressing (Steri-Strip) was applied to all the noses after surgery to minimize soft tissue swelling and graft displacement. Patients who had osteotomy had additional nasal splints applied to the nasal dorsum for about a week. The average follow-up duration was 16.9 months, and the length of stay in the hospital was 4.6 days (range 3–7 days).

The patients were followed up postoperatively. The removal of the transcolumellar sutures, Steri-strip, and nasal splint was performed on the 5th postoperative day. On followup, the patients were reviewed with respect to their improvement in symptom scores for nasal patency and aesthetic improvement. While the patient's satisfaction was entirely subjective, nasal endoscopy was performed to evaluate patency of the nasal airway. Presently, 20 patients are on active followup with a mean duration of 15 months, while 6 defaulted at an average of three months. One patient had intranasal synechiae formation which was released later at followup in clinic using local anaesthesia. Two patients had transient tip paresthesia with minimal alar depression. Another two patients had alar deformity which required revision surgery. There has been 3 cases of nasal infection, epistaxis, and implant extrusion. Functionally, all except one patient experienced subjective improvement in nasal airway. Thus, the postoperative nasal obstruction rate was one in twenty (5.9%) patients.

The scale of the patient's level of satisfaction was subjective. Among those still on active followup, 16 of 20 (80%) patients were satisfied with the cosmetic improvement. However, two patients with minimal alar depression were moderately satisfied. Two patients had obvious alar deformities and agreed to undergo revision surgery. After the revision surgery, one patient was moderately satisfied whereas the other patient was still dissatisfied with the looks. These patients remained on followup for possible revision surgery in the near future.

## 4. Discussion 

The cartilaginous septum and the maxillary crest bone form the main support of the lower two-thirds of the nasal dorsum, and if there is insufficient cartilage to give support either due to absence or fibrosis of the cartilaginous part of the septum, nasal saddling to various degree will result [4]. Nasal saddling is therefore commonly seen after septal haematoma, septal surgery, or trauma, and if haematoma is infected, nasal collapse is almost inevitable. Grafting of the dorsum is deferred until the degree of saddling is evident. Loss of septal support for the nasal dorsum may occur in chronic inflammatory conditions which involve cartilage such as sarcoidosis, tuberculosis, and syphilis. Some degree of saddling may also be familial or racial in characteristic.

The internal nasal valve is an important anatomical landmark that must not be overlooked in the preoperative assessment. It is bordered medially by the septum, inferiorly by the nasal floor, laterally by the inferior turbinate, and superiorly by the caudal border of the ULC. Any compromise in the surrounding boundaries would render the valve susceptible to collapse, resulting in nasal obstruction. Once the boundary of the nasal valve is interfered, repair by autogenous grafts or allografts is essential if patient is symptomatic.

Proper and standardized preoperative and postoperative photography is essential in rhinoplasty for medical record and for medicolegal purpose [5].

Rhinoplasty cannot be successfully undertaken until the major and minor nasal tip support mechanisms are appreciated, respected, and preserved (Table 3). Loss of tip support and projection (tip ptosis) in postoperative healing period is one of the most common surgical errors in rhinoplasty.

The projection and rotation of the nasal tip may be conceptualized through Anderson's tripod mechanism [6], which the author believes is more appropriate for Caucasian noses. The two lateral crura and the conjoined medial crura create the three supporting limbs of the tripod. Therefore, shortening the medial crura will counterrotate and deproject the tip; lengthening the medial crura will rotate and project; shortening the lateral crura will rotate and deproject; and lengthening the lateral crura will counterrotate and project. 

Immediate changes to the tripod mechanism may be performed through a combination of repositioning techniques such as suture retropositioning the medial crura onto the caudal septum in order to decrease projection and rotation; modification of structural shape such as dome suturing to increase projection; structural grafting such as tip grafting to increase projection; or overlapping techniques such as lateral crural overlay to deproject and increase rotation. It is preferable to avoid excessive reduction, excision, or weakening of tip structures.

Trauma accounted for most cases of twisted nose [7, 8] with other cases being congenital or prior to nasal surgery. Besides the obvious cosmetic defect, patients with twisted nose frequently have troublesome nasal obstruction due to narrowed airway.

The twisted nose comprises distortion of the mid-vault osteocartilaginous framework in various possible combinations with majority of patients having significant deviated septum [8]. The objective of the surgery is to achieve or restore a straight, midline, and supportive septum. It is important to maintain an adequate L-shaped dorsal strut of the remaining quadrangular cartilage (1–1.5 cm) at all times to preserve the dorsal and caudal support. Often, grafts (autograft or allograft) are needed to resist the memory effect and prevent recurrence of the curved septum in these groups of patients [7]. A septum dislocated off the maxillary crest should be repositioned. Therefore, history of allergic rhinitis should be elicited, and proper medical management commenced before the definitive corrective functional surgery. Moreover, this will allow the surgeon to determine the severity of the functional problems contributed by the structural defect.

## 5. Anatomical Divisions of the External Nose

One can divide the external nose structure into thirds for simplicity of understanding of the osteocartilaginous framework.

### 5.1. Upper Third

 The upper third comprises the nasal bones and extends down to the osteocartilaginous junction called the rhinion.

### 5.2. Middle Third

The middle third (mid-nasal vault) is made up of the upper lateral cartilages (ULCs) and septum. Involvement of the middle third in twisted or saddled noses is more difficult to correct due to the inherent tension memory effect of the ULCs and septum. The ULC need to be mobilized away from the septum keeping the mucoperichondrium intact to maintain the vascularity to the mobilized ULC and prevent scarring in the nasal valve region [5, 7]. Furthermore,, when separating the ULC from the dorsal septum, it can be difficult to create a straight medial margin without resecting even more ULC, thus making a spreader graft mandatory [5].

### 5.3. Lower Third

The lower third comprises the lower lateral cartilages (LLCs), the anterior septal angle, and caudal septum. Deformity of the lower third is usually caused by deformity in the caudal septum or the nasal tip. If the nasal valve area is not compromised here, the use of spreader graft may not be necessary. Most twisted noses have some form of anatomic distortion of the lower two-thirds [6].

## 6. Open Approach

An open approach would allow direct visualization of the tip problem and subsequent tip plasty under direct vision. The external or open approach is essentially a more aggressive form of delivery. When the nasal tip is highly asymmetrical, markedly overprojected, severely underprojected, or anatomicaliy distorted as in secondary revision cases, the open approach is considered. The transcolumellar scar is of negligible significance as it routinely heals inconspicuously when meticulously repaired. The anatomical view is unparalleled through this approach, affording the surgeon diagnostic information unavailable through traditional closed approaches. These technical virtues must be balanced with potential disadvantages of enlarged scar bed, slightly delayed healing with prolongation of tip oedema, and increased operating time. When subtle and conservative tip surgery is indicated by the patient's existent anatomy, the open approach is unnecessary and even counterproductive.

### 6.1. Indications for Choosing the Open Approach

These include the following:

1. asymmetrical tip cartilages,
2. severe tip underprojection or overprojection,
3. severely deviated nose,
4. middle vault deformities requiring grafting,
5. nasal tumors [9],
6. cleft lip/nose deformities,
7. difficult revision rhinoplasty,
8. infantile nostrils,
9. a teaching tool.

## 7. Graft Selection

There are a wide variety of graft materials available for nasal augmentation which are successfully used. Each portion of the nose has different characteristics that may require different augmentation material. Establishing a good rapport with the patient can help increase the potential for satisfaction with the postoperative result.

## 8. Autologous Graft Selection

While autogenous grafts are the gold standard for augmentation in open septorhinoplasty, allograft can still be used for well-selected patients. The patient should be counseled regarding the available options for the graft material to be harvested. Autogenous materials incite much less inflammatory response with low rates of resorption, extrusion, and infection, though it may be associated with donor site morbidity and longer operating time. More often, autogenous grafts are used for reconstruction.

### 8.1. Quadrangular Cartilage Graft

The gateway to the nose for the otorhinolaryngologist is the septum. The quadrangular cartilage and bone septum is a useful supply of cartilage and bone that is easily harvested during surgery [6]. There have been reported cases where the rostrum has been harvested from the sphenoid bone in addition to septal cartilage to reconstruct the nasal dorsum [7]. Quadrangular cartilage grafts are harvested and subsequently trimmed and shaped according to the size of the defect and strength of support needed. Using quadrangular septal cartilage is advantageous to the otorhinolaryngologist because it is locally available in the same surgical field and because of the ease of contouring the cartilage. If quadrangular septal cartilage is inadequate, the conchal cartilage can be harvested instead. In midthird external deformity, the spreader graft can then be placed unilaterally or bilaterally between the upper lateral cartilage and the septum and stitched with prolene 5/0 suture fixation. Other alternatives described include placing the graft in mucoperichondrial pockets with no suture fixation, but both methods were equally effective [10]. There were 14 cases in this study where quadrangular cartilage spreader graft was utilized.

### 8.2. Conchal Cartilage Graft

Another source of harvested graft can be from the pinna, where the conchal cartilage is pliable and easily shaped [5]. An essential consideration in harvesting conchal cartilage is to use portions of the concha that are most similar to the nasal anatomy. The cymba concha due to its curvature is suitable for reconstruction of the lateral crura and correction of saddled nose deformity [11]. The cavum concha which is thicker and stiffer is ideal for projection of the nasal tip [12, 13]. Allografts can be considered if there is inadequate cartilage graft available; however, first consideration should be given to autogenous material. An onlay graft is essentially useful in cases where the ULC or nasal bones are depressed without associated airway problem [7]. Conchal cartilage is easily available, easy to carve, having low donor site morbidity, and less metabolically demanding; thus it undergoes less resorption [14]. There was one case in this study where conchal cartilage graft was utilized.

### 8.3. Costal Cartilage Graft

Costal cartilage is becoming an increasingly more common graft source, particularly in secondary rhinoplasty, because it can provide a relatively large source of autogenous material. The use of costal cartilage grafts for facial reconstruction is challenging because harvested costal cartilage tends to warp over time. Warping is unacceptable in facial reconstructive surgery, particularly in rhinoplasty, because even slight changes in postoperative graft shape may lead to noticeable deformity or functional deficiency. In 1920, Gilles [15] noted that grafts carved from the periphery of costal cartilage sections tend to warp toward the more peripheral side. Gibson and Davis [16] theorized that the immediate and delayed warping of costal cartilage grafts is due to inherently increased tautness in peripheral regions and therefore advocated cutting balanced cross sections to minimize these forces. The concept of balanced cross sections to prevent warping facilitates that use of costal cartilage in reconstructive surgery. Even though it is accepted that central slices warp significantly less than peripheral slices, in a practical setting, grafts harvested from central regions are not completely resistant to warping [17]. Costal cartilage, although it has been recommended for more extensive deformities, has the disadvantage of incurring significant donor site morbidity and reported incidence of warping [13, 14].

Spreader graft was designed to address the aesthetics of the dorsal lines and the problem of nasal valve collapsing with subsequent nasal obstruction [11]. The graft acts to widen the nasal valve angle and significantly improve the nasal airway. The Literature reviews highlight that spreader grafts were found to be most successful in correcting severe middle vault deformity and internal nasal valve related airway problems [12, 13]. In patients with both twisted nose and prominent dorsal hump, spreader graft was used along with columella strut in an attempt to prevent future vestibular contraction [11].

There were 3 cases in this study where rib cartilage graft was utilized either in the form of spreader graft, dorsal graft, columella graft, shield graft, or batten graft.

### 8.4. Bone Graft

Bone grafts are used in patients where more rigid augmentation was needed. Septal bone was easily available within the surgical field from either the bony septum, sphenoid rostrum [8], or the maxillary crest. Iliac crest bone provides ample supply of relative flat bone, but is associated with donor site morbidity, namely, pain and hematoma. On the hind side, the rigidity of bone grafts makes them less suitable for areas like the nasal tip. There were two cases of septal bone graft utilization in this study with no evidence of iliac bone graft harvest.

## 9. Synthetic Graft Selection

Synthetic grafts have the advantage of being in abundant supply and reduce operating time and donor site morbidity. History of previous surgery or patient's apprehension about increased surgical morbidity from a second operative site may contribute to the surgeon's decision to use a nonautogenous graft [18]. In the literature, other than Medpore, other materials that have been used include expanded polytetrafluoroethylene (Gore-Tex, WL. Gore and Associates) and dimethylsiloxane polymer (Silastic, Dow Corning Corp).

### 9.1. Medpore Graft

Medpore allograft offers some advantages in nasal reconstruction. The internal pores of varying sizes facilitate fibrous and vascular tissue ingrowth, therefore allowing mechanical stabilization with less risk for infection and extrusion. It is easily available locally in numerous shapes and sizes and is readily sculpted after being soaked in hot water. Besides being malleable, it incites minimal foreign body reaction as compared to silicone implant [14]. There were 8 cases in this study where Medpore was utilized as spreader graft, columellar graft, or dorsal support graft.

### 9.2. Silicon Graft

Silastic, a silicone-based implant, is avoided, as it is notorious for the high rates of infection and extrusion, especially in thin-skinned individuals [19], due to lack of fibrous or vascular ingrowth into the implant. Other complications like graft migration and dorsal cyst formation have also been documented [20]. There was no evidence of silicon graft use in this study.

### 9.3. Gore-Tex Graft

Gore-Tex, composed of fibrillated polytetrafluoroethylene (PTFE), shares common advantages as those of Medpor. It is highly biocompatible allowing tissue ingrowth with minimal inflammatory response and low rates of infection, extrusion, and resorption. However, with its soft consistency, it does not provide a robust structural integrity for augmentation [14]. There was no evidence of Gore-Tex utilization in this study.

## 10. Multiethnicity

Multiethnicity in Malaysia is also a factor to be taken into consideration during septorhinoplasty, as it significantly affects the surgical techniques used and the eventual outcome. Indians tend to possess the nasal geometry of the Caucasians compared to the Malay or Chinese (Oriental) features due to their ancestral roots. Caucasian Indian nose is more highly projected at the tip and nasion with less prominent alar flare as compared to the Oriental Malay and Chinese nose which has a less prominent nasal dorsum and tip projection with a prominent alar flare [21, 22]. On the other hand, non-Caucasian noses have thicker skin, weak cartilage, flat broad dorsum with underprojected tip, wider alar base, and shorter nasal bones [23]. Thickness of the skin affects the prominence of the underlying cartilages, ease of tissue dissection, and the degree of nasal tip sculpting. The transcolumella incision should also be placed lower in non-Caucasian noses because augmentation will enhance the columella skin cephalically [23].

## 11. Complications

Foda [24], in his critical analysis of his rhinoplasty experience, documented his complication rates as follows: septal flap tear 2.8%, alar cartilage injury 1.8%, postoperative nasal trauma 1%, epistaxis 2%, infection 2.4%, prolonged edema 17%, nasal obstruction 0.8%, and unsightly transcolumellar scar 0.8%. The overall patient satisfaction rate in his series was 95.6%. In this series, while the complication rate for residual deformity was higher, there has been no cases of postoperative epistaxis, infection, graft extrusion, or keloid scar formation either at transcolumellar or rib cartilage incision site.

## 12. Conclusion

Septorhinoplasty continues to evolve through various new techniques and modifications with the main goal to improve functional nasal airway and to restore cosmetic harmony to the face. Optimum result is very much dependent on the surgeon's attention to functional, aesthetic, and reconstructive principles and graft selection. Performing rhinoplasty in a multi-ethnic community possesses more challenges to the rhinoplastic surgeon. Moreover, the best intentions and efforts for the betterment of the patients must be balanced by the surgeon's initiative to keep improving his clinical acumen and surgical skills. 

## Figures and Tables

**Table 1:** Demographic data of 26 patients who underwent open rhinoplasty.

Patient	Age/sex/race	Trauma	Deformity	Graft/Implant	Hospitalization (days)	Complications	Followup (months)
1	16/F/M	Yes	Crooked nose	QCSG	4	—	9
2	24/F/I	Yes	Twisted nose	QCSG	4	Tip paresthesia Depressed alar	40
3	24/M/C	Yes	Twisted nose	SBG	4	Unilateral nasal obstruction	3
4	35/M/C	Yes	Saddled nose	SBG	4	—	3
5	37/M/C	No	Crooked nose	QCSG	5	—	32
6	19/F/I	No	Crooked nose	QCSG	4	Alar deformity	32
7	38/M/I	Yes	Twisted nose	QCSG	5	Tip paresthesia Alar deformity	31
8	16/M/I	No	Twisted nose	MSG	4	Synechia	23
9	44/M/I	No	Underprojected nasal tip	MSG + MCG		—	22
10	16/M/I	No	Twisted nose	MSG	5	—	20
11	26/F/I	No	Twisted nose	QCSG	4	—	20
12	32/M/I	No	Twisted nose	QCSG	4	—	9
13	20/F/I	No	Crooked nose	QCSG	5	—	3
14	22/F/I	No	Saddled nose	MSG	4	—	15
15	16/M/I	Yes	Twisted nose	QCSG	5	—	14
16	46/F/I	Yes	Saddled nose	MDSG + CCDG	5	Alar deformity	14
17	45/M/I	No	Twisted nose	QCSG	6	—	12
18	54/F/I	No	Twisted nose	QCSG	4	—	12
19	40/M/I	No	Dorsal hump	MSG	5	—	11
20	22/M/M	No	Twisted nose	QCSG	6	—	10
21	38/M/I	No	Twisted nose + dorsal hump	MDSG + QCDG	7	—	9
22	35/M/C	No	Twisted nose	MSG	5	—	9
23	22/M/M	Yes	Twisted nose	MDSG + QCDG	3	—	9
24	32/M/I	No	Twisted nose	RCSG + RCShG + RCDG	5	—	26
25	22/M/C	No	Underprojected nasal tip	RCDG + RCCG + RCShG	4	Stitch abscess	19
26	54/M/I	No	Repaired cleft/palate with under Projected nasal tip and alar asymmetry	RCDG + RCCG + RCShG + RCBG	5	— —	21 13

QCSG: Quadrangular cartilage spreader graft.

SBG: Septal bone graft.

MSG: Medpore spreader graft.

MCG: Medpore columellar graft.

MDSG: Medpore dorsal support graft.

CCSG: Conchal cartilage spreader graft.

CCDG: Conchal cartilage dorsal graft.

RCSG: Rib cartilage spreader graft.

RCDG: Rib cartilage dorsal graft.

RCCG: Rib cartilage columellar graft.

RCShG: Rib cartilage shield graft.

RCBG: Rib cartilage batten graft.

**Table 2:** Types of grafts used for various nasal deformities.

Deformities	Grafts	Amount
Crooked nose	QCSG	4
Dorsal hump	MSG	1
	SBG	1
	QCSG	8
Twisted nose	MSG	3
	MDSG + QCDG	1
	RCSG + RCShG + RCDG	1
Twisted nose and dorsal hump	MDSG + QCDG	1
	MSG	1
	MSG + CCSG	1
Saddled nose	SBG	1
Underprojected tip	MSG + MCG	1
	RCDG + RCCG + RCShG	1
Repaired cleft lip/palate with underprojected nasal tip and alar asymmetry	RCDG + RCCG + RCShG + RCBG	1

**Table 3:** Nasal tip support mechanism.

*Major supports of the nasal tip *	
(1) Size, shape, thickness, and resilience of alar cartilages	
(2) Upper lateral cartilage attachment to the cephalic margin of the alar cartilages	
(3) Wraparound attachment of the medial crural footplates to the caudal septum	

*Minor supports of the nasal tip *	
(1) Anterior septal angle	
(2) Skin and nasal tip	
(3) Membranous septum	
(4) Caudal septum	
(5) Nasal spine	
(6) Ligamentous sling spanning the paired domes of the ala cartilages	
(7) Sesamoid cartilage complex extending the support of the lateral crura to the pyriform margin	
